# Finding relevant free-text radiology reports at scale with IBM Watson Content Analytics: a feasibility study in the UK NHS

**DOI:** 10.1186/s13326-019-0213-5

**Published:** 2019-11-12

**Authors:** Alicja Piotrkowicz, Owen Johnson, Geoff Hall

**Affiliations:** 1 Leeds Institute of Medical Education, University of Leeds, Leeds, UK; 2 Leeds Teaching Hospitals Trust, Leeds, UK; 3 School of Computing, University of Leeds, Leeds, UK; 4 School of Medicine, University of Leeds, Leeds, UK

**Keywords:** Information retrieval, Natural language processing, Radiology, Feasibility study, Rule-based system

## Abstract

**Background:**

Significant amounts of health data are stored as free-text within clinical reports, letters, discharge summaries and notes. Busy clinicians have limited time to read such large amounts of free-text and are at risk of information overload and consequently missing information vital to patient care. Automatically identifying relevant information at the point of care has the potential to reduce these risks but represents a considerable research challenge. One software solution that has been proposed in industry is the IBM Watson analytics suite which includes rule-based analytics capable of processing large document collections at scale.

**Results:**

In this paper we present an overview of IBM Watson Content Analytics and a feasibility study using Content Analytics with a large-scale corpus of clinical free-text reports within a UK National Health Service (NHS) context. We created dictionaries and rules for identifying positive incidence of hydronephrosis and brain metastasis from 5.6 m radiology reports and were able to achieve 94% precision, 95% recall and 89% precision, 94% recall respectively on a sample of manually annotated reports. With minor changes for US English we applied the same rule set to an open access corpus of 0.5 m radiology reports from a US hospital and achieved 93% precision, 94% recall and 84% precision, 88% recall respectively.

**Conclusions:**

We were able to implement IBM Watson within a UK NHS context and demonstrate effective results that could provide clinicians with an automatic safety net which highlights clinically important information within free-text documents. Our results suggest that currently available technologies such as IBM Watson Content Analytics already have the potential to address information overload and improve clinical safety and that solutions developed in one hospital and country may be transportable to different hospitals and countries. Our study was limited to exploring technical aspects of the feasibility of one industry solution and we recognise that healthcare text analytics research is a fast-moving field. That said, we believe our study suggests that text analytics is sufficiently advanced to be implemented within industry solutions that can improve clinical safety.

## Background

A wealth of healthcare data is stored in unstructured free-text reports [1]. These include narrative reports from diagnostic services such as radiology and pathology, consultants’ letters, discharge summaries and clinical notes. For example, Capurro et al. [2] found that in two US health IT systems only about 25% of the data required to phenotype patients was available in a structured and easily extractable format. Clinicians, researchers, and administrators often need to retrieve and read these clinical reports to understand the details of patient care. For busy clinicians with limited time the requirement to read large amounts of free-text presents a risk of information overload and consequently missing information vital to the care of their patients. In the past such documents were often handwritten and stored on paper but in parallel with advances in electronic health records (EHR) many of these free text documents are now generated digitally and stored within, or linked to, the EHR [1].

Automatically identifying critical information at the point of care has the potential to reduce the clinical risks associated with missing this information but also represents a considerable challenge [3]. The current range of commercially available tools such as IBM Watson claim to offer support that will improve clinical outcomes through more efficient and accurate document search and text analytics. More generally, there has been considerable research effort to create systems which can reliably identify and extract relevant information from clinical text. Implementing such systems at scale in a clinical environment is difficult. As we see it, the challenge is twofold. Firstly, from the technological perspective we need reliable and accurate means of automatically extracting information using natural language processing methods, and this automatic extraction needs to happen at scale and be readily available for querying. Secondly, from an operational perspective, such a text analytics system would need to be integrated into clinical practice in a way that preserves data security and delivers information in real-time at point of care.

In this paper we present a feasibility study of implementing one specific commercial text processing system, IBM Watson Content Analytics,^1^ using data from the UK National Health Service (NHS). IBM Watson Content Analytics provides an architectural framework for loading, querying, and searching free-text documents at scale. The Content Analytics suite belongs to the family of IBM Watson products, however unlike some of the other products such as IBM Watson Developer Cloud,^2^ this is a system which can be installed locally. Patients are rightly concerned that their personal medical data does not leave the security of their healthcare provider and local installation is seen as crucial to supporting the data protection policies that safeguard clinical data. In previous work we have argued that this is especially important for free text where the risk of disclosure of sensitive information within the text is ever present [4]. We designed [4] and then implemented the Integrated Research Campus (IRC) [5], a large scale infrastructure for health data analytics to help address these concerns.

### Aim

The aim of our feasibility study was to understand the potential contributions that a commercial text analytics system such as IBM Watson Content Analytics could make within Leeds Teaching Hospitals Trust (LTHT), a large UK NHS hospital. We adopted a case study approach and used IBM Watson to find all radiology reports within the hospital database with the incidence of two conditions: hydronephrosis and brain metastasis, defined as those reports that indicate a positive diagnosis of one of the two conditions (for example we exclude positive mentions from clinical history). We hoped to use this study to identify the range of institutional, technical, and algorithmic factors which could influence the implementation of clinical text analytics more widely in the NHS. To understand the generalisability we used the same architecture on a similar set of radiology reports extracted from the MIMIC-III open access dataset which comes from the Beth Israel Deaconess Hospital in Boston, USA - a different hospital in a different country with a very different healthcare system [6]. For this study we agreed that we would not attempt to benchmark IBM Watson Content Analytics against alternative tools or conduct a financial evaluation of its costs and benefits.

### Project setup

This feasibility study took place over a three-month period in 2016. The person commitment included one full-time PhD student in computer science with an honorary NHS contract for the duration of the project, an IBM Watson training expert for 1 week, and part-time commitment from senior academics, including a clinical expert. Our IRC information security management infrastructure is ISO 27001 certified and provides secure data storage and processing facilities in a research environment directly connected to LTHT. Datasets were transferred in anonymised formats under appropriate ethics and information governance frameworks, and within the IRC data safe facilities. Access was restricted to NHS staff located within the secure facilities using a secure Citrix connection to an audited, secure Virtual Machine running within the IRC on which we installed IBM Watson Content Analytics.

### Limitations

There were several limitations to our study. Firstly, we use a case study in a research environment connected to clinical systems, instead of directly within a live clinical system. Secondly, we used historical data, not a live stream. Thirdly, our case study was limited to a small number of report types and clinical conditions, whereas an actual implementation would be a part of a larger pipeline.

### Contributions

Our contributions are as follows: (i) we present an implementation of a large-scale commercial text analytics system which uses NHS data, (ii) we present an overview of IBM Watson Content Analytics and the results of a case study in the radiology domain, and (iii) we show that a task-specific model generalises across radiology reports in two different countries (US and UK) for the two conditions we chose.

### Information retrieval (IR) approaches

Some of the existing clinical search systems resemble commonly used search engines like Google. They share some traits with most IR systems, such as relying on string matching and using indexing for quick retrieval of documents from a large repository. Examples include STRIDE [7] and CISearch [8]. One issue encountered in the clinical domain is that the same clinical concept can be expressed by a wide variety of lexical forms and their acronyms. In the case of search engines that requires submitting multiple queries in order to retrieve as many relevant documents as possible. Hanauer et al. [9] addressed this by already identifying UMLS concepts in text (which allows searching concepts, not just lexical forms), as well as providing the facility to create ‘search bundles’ which allow to group appropriate search terms. The advantage of keyword-based clinical IR systems is their generalisability – since the indexing works on a token (i.e. individual word) level (more rarely concept level), any type of clinical report can be processed by the system. However, the downside of using keyword matching without looking at the context is that in many cases irrelevant documents are returned (e.g. when the relevant keyword is negated). Taking context into consideration requires a deeper linguistic analysis, which is why there is an increasing drive to use and develop NLP methods with clinical text.

### Natural language processing (NLP) approaches

There have been a significant number of research efforts in the domain of natural language processing of clinical text. An example of continuing research interest in this domain is the inclusion of a biomedical and clinical text analysis task in the i2b2 [3] and SemEval (Semantic Evaluation) challenges, which are a widely used evaluation and benchmarking systems in the field of natural language processing.^3^ The majority of NLP architectures to date have relied on a combination of lexical dictionaries and rules. More recently, supervised machine learning approaches have also been used.

Many of these clinical NLP tasks concern themselves with the automatic annotation of clinical text, which goes further than simple string matching of relevant lexical forms from medical ontologies. These approaches need to address the challenges of clinical text annotation including misspellings, context-sensitive abbreviations, and negation. Some of these are already handled by more sophisticated IR systems (e.g. handling of misspellings by Hanauer et al. [9]). ConText [10] is an example of an NLP system that employs shallow, surface-based methods to annotate a variety of linguistic aspects in clinical documents. Specifically, ConText evaluates whether a mention of a clinical condition is negated, hypothetical, historical, or experienced by someone other than the patient. This is done by matching certain keywords in text by using dictionaries and using rules to infer the relation between matched keywords. This approach of using dictionaries and rules is quite common for clinical NLP tasks. Unlike machine learning approaches where patterns are learnt from the data, rule-based systems are preferred because they provide decision provenance [11]. Furthermore, training of supervised machine learning models requires considerable manual effort to provide annotations [12]. However, an annotated dataset could be reused for training different machine learning models, as well as different tasks (e.g. entity recognition, coreference resolution).

### Hybrid (NLP and IR) systems

Hybrid systems combine the strengths of IR and NLP approaches to allow accurate processing of clinical documents at scale. One of the most successful being the caTIES system [13] which employs a query-based architecture to process pathology reports within a live clinical setting. cTAKES [14] is an open-source toolkit for annotating clinical documents that can be customised by adding relevant dictionaries.

### IBM Watson in healthcare

IBM Watson is one of a number of commercially available cognitive computing products that are exciting popular interest in the transformative potential of artificial intelligence and big data [15], including in the UK NHS [16]. Despite the interest, there is relatively little academic literature on IBM Watson and its application to healthcare. The most widely cited example of IBM Watson in healthcare is IBM Watson for Oncology [17, 18] developed with Memorial Sloan Kettering hospital in the USA [19] and focused on providing clinical decision support by matching patient conditions to those in scientific oncology publications using NLP. The more widely available NLP tool within the IBM Watson product set is IBM Watson Content Analytics. This has been used for dermatology [20] and patient safety research and risk management [21]. In the UK, IBM Watson is being used on patient generated text at Alder Hey NHS Trust [22] but as far as we are aware this paper is the first to explore the possibility of implementing IBM Watson on clinical free text in the NHS. In addition, we extended our work to an open access corpus from the USA to explore the generalisability of our rule-based approach to other hospitals and English speaking countries.

## Overview of IBM Watson Content Analytics

**Fig. 1:**
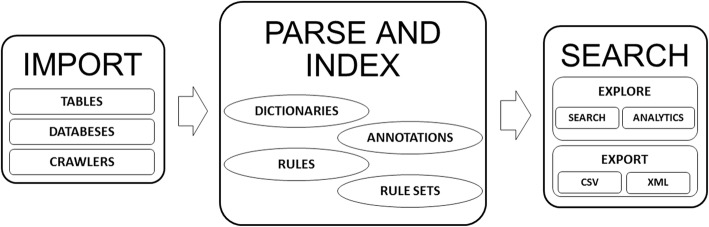
Overview of IBM Watson Content Analytics

We present an overview of the IBM Watson Content Analytics system in Fig. 1.

The system consists of three main components, which (i) import, (ii) parse and index, and (iii) search documents. The Unstructured Information Management Architecture (UIMA) [23] is implemented throughout the system and allows for adding of further components into the pipeline. We will now describe each component in more detail.

### Import

The importing of documents can be done as a one-off task (ingestion), or continuously using a crawler in a specified directory. A variety of data formats are handled, including flat tables, databases, and XML.

### Parse and index

The raw documents are first processed. Built-in methods separate the documents into smaller chunks (paragraphs, sentences, tokens). Like in standard IR systems, this system relies on matching keywords against document text. Keywords can be grouped into **dictionaries**. For example, any words or phrases describing cancer can be grouped into a *Cancer* dictionary. Existing dictionaries can also be uploaded. Dictionary matches in text (optionally with surrounding tokens) can be turned into **annotation**. Each annotations can be tagged with additional information, e.g. length = n characters, word category = noun. Annotations can be combined with context information (e.g. tokens or other annotations) into **rules**. Rule building uses a syntax similar to regular expressions, including grouping and repeats. The order of annotations or tokens in a group can be controlled by using ordered or disordered groups. Rules can also act on different levels of the document, e.g. tokens, sentences, or the whole document. For example, a simple rule for annotating a mention of cancer location could say that within a single sentence there should be an annotation for a body organ and an annotation for a cancer keyword (both annotations identified using dictionaries) with at most five tokens in between them. This would match *breast cancer,* and *cancer has metastasized to the brain.* Finally, individual rules can be combined into **rule sets**, which perform a specific language task, e.g. Negation. Finally, any annotations can be turned into ‘facets’, i.e. searchable indices.

### Search

Once the documents are processed, they can be searched using the index. The use of the index allows for a quick and efficient searching through very large document collections. Specific annotations (e.g. Age, Gender, Condition) from the parsing stage can be used as ‘facets’. These facets can be used to search and filter documents. A helpful feature is the search results view with text snippets with the facets highlighted in different colors, which allows an at-a-glance overview of results without having to open every document separately. Facets can also be combined. For example, first select all documents which mention a specific condition, then add the Gender facet. Depending on the contents and processing of the document some further analytics (e.g. time series) are also available.

## Results

In this section we present the results of our investigation using IBM Watson Content Analytics for processing the free-text clinical reports in our case study. We used 5.6 m radiology reports from LTHT in the UK and repeated our experiments using 0.5 m radiology reports from similar set of radiology reports extracted from the MIMIC-III dataset from the USA [6]. MIMIC-III is an open access dataset and can therefore be used by other researchers interested in reproducing or improving on our approach.

### Task definition

Our goal is to identify all clinical reports with a positive incidence of a particular condition. By positive instance we mean that the condition is diagnosed or identified in the report (‘X is noted’), and negated instances (‘without X’) or historical instances (‘on admission the patient presented with X’) should be excluded.

### Conditions

We chose two conditions for the case study: (i) hydronephrosis (swelling of a kidney), and (ii) brain metastasis (cancer cells which have spread to the brain from another primary tumour). By looking at two different conditions we can investigate to what extent the resources and models for one condition generalize to another (i.e. which resources can be reused for different conditions and which have to be adjusted or developed from scratch).

### Data

We used two different radiology datasets. Our primary dataset were the radiology reports from Leeds Patient Pathway Manager (Leeds PPM), the EHR system used by LTHT [24]. We were also interested in using a more widely accessible clinical dataset, which would allow us to compare methods. Sharing data in the healthcare domain has been a longstanding issue due to the highly sensitive nature of health records and this is especially the case for clinical reports, since removing identifiable patient information from text is complex and time consuming. The MIMIC-III dataset^4^ [6] is therefore a very valuable research resource in that it provides researchers access (subject to conditions) to a large number of English language free text clinical documents linked to a full set of EHR records and already de-identified and prepared for research use. An overview of the two datasets is presented in Table 1.

**Table 1:** Summary of the two radiology data sources

	LTHT PPM	MIMIC-III
EHR data source	Leeds Teaching Hospitals NHS Trust, Leeds, UK	Beth Israel Deaconess, Boston, MA, USA
Period	1996–2016	2001–2012
# Patients	~ 1 million	~ 37,000
# Documents	~ 5.6 million	~ 0.5 million
Av. report length	70 tokens	206 tokens

### Methods

The following metaprocess was used in our pilot study.

1. Set up IBM Watson Content Analytics: prepare the server, training and familiarization
2. Obtain datasets and prepare overview
3. Initial processing: segment all documents into tokens which allows for a simple keyword search
4. Data exploration: using keyword search find concordances of a condition and study the context
5. Create a random sample of 1000 documents for in-depth analysis and initial development
6. Iterative development of a model: creation of dictionaries and rule sets
7. Initial evaluation: evaluate precision (i.e. are the annotations and rules correct) using the Watson analytics and highlights features
8. Extend model to a bigger sample, and then to the full dataset
9. Evaluation against a manually annotated gold standard

### Dictionaries

Dictionaries of clinical terms for the two conditions (hydronephrosis and brain metastasis) were created by a medical domain expert. Potential additions found in the corpus during model development were consulted. We developed separate dictionaries of negative instance indicators: negation (e.g. *no*, *without*), evaluation (e.g. *query*, *evaluate*), clinical history (e.g. *admitted*, *history*), resolution (e.g. *resolved*, *cleared*). These dictionaries were considerably expanded during the iterative development process by analysing the false positive cases.

### Rules

The general design behind the rules for this task is that a condition mention is presumed a positive instance, unless explicitly negated using a negative instance indicator. We now present a simplified rule set for hydronephrosis.

Rule set for hydronephrosis (cohesive ConditionKeyword):

1. Create annotations from dictionaries: ConditionKeyword, EvaluationIndicator, NegationIndicator, HistoryIndicator, ResolutionIndicator. Additional dictionaries (e.g. Age, Gender, ICD-9 codes) can be added to use for analytics in data exploration and search stage.
2. For the ConditionKeyword annotation, create some indication of condition incidence, e.g. present = unknown, polarity = unknown
3. For each negative instance indicator dictionary (evaluation, negation, history, resolution), create the following rules, where [S …] indicates that the scope of the rule is a sentence:
  - If [S NegIndicator ConditionKeyword] then ConditionKeyword/Presence = false
  - If using an ordered group then optionally depending on data: [S ConditionKeyword FalseInd]
4. For any other instance of ConditionKeyword, ConditionKeyword/Presence = true
5. Optional: add lastOccurrence = unknown to ConditionKeyword annotation and set to true/false. In the evaluation consider only true instances if they are also the last occurrence of the ConditionKeyword in the document (this helps with eliminating historical incidence and ambiguous cases)

### Extending the hydronephrosis model to brain metastasis

The main difference between the hydronephrosis and brain metastasis case studies are the keywords indicating the condition. Firstly, the dictionary of hydronephrosis keywords is (as far as we know) complete for our case study and these keywords point unambiguously at this particular condition. Furthermore, in cases of multiword expressions (e.g. ‘dilated renal pelvis’), the words occur together, so simple string matching is sufficient to create annotations. However, for the brain metastasis case the condition keywords are sometimes non-contiguous (e.g. “Within the *brain* multiple ring enhancing *metastatic* deposits noted”). As they occur separately, two different dictionaries need to be built (Dict1 = brain-related keywords, Dict2 = metastasis-indicating keywords), and what follows is that the annotation needs to be built on the sentence level. Secondly, both brain metastasis dictionaries have some issues. The brain-indicating keywords can be ambiguous. Some keywords refer to different body parts (e.g. parenchymal, lobe) and it is sometimes difficult to tell definitely just from the report text (however the report title can be help in disambiguating, e.g. CT Head will point to brain parenchyma, but CT Abdomen would not). The metastasis-indicating keywords can be quite generic (e.g. *tumour* which can indicate both primary and metastatic tumours). For example, if brain metastasis is queried explicitly, then in the findings section the radiologist might not say “brain mets found”, but rather “multiple lesions found”. Because of the context a human will be able to tell that this means metastatic lesions in the brain, but it is challenging to implement computationally because of the long-range reference. The long-range reference is also an issue when inferring the location of the tumour (e.g. “There are two enhancing *lesions* with a moderate degree of surrounding oedema. The largest measures 2.7 cm and is in the left *frontal lobe*. The second measures 1.5 cm and is located in the right *occipital lobe*.”). While the models for the two conditions differ, some elements (e.g. negative instance dictionaries) can be effectively reused, saving development time.

### Extending the model to a different dataset

While each condition required a customised model, we found that once models had been developed they were effective on both the data from the UK and from the USA, two very different hospitals, countries and healthcare environments. We believe this is because there is a significant overlap in medical training and terminology across the English speaking world, especially in the case of technical clinical reports such as those written in radiology. We acknowledge that despite similarities in jargon, there are still country- or institution-specific conventions (e.g. use of certain acronyms) and differences of spelling between UK English (e.g. *tumour*) and US English (e.g. *tumor*). Some conventions are more localised (e.g. frequent use of *assess* for Report Indication in the US dataset), but these were easily resolved by adding some new terms to the relevant dictionary. This addresses the point made by Pons et al. [25] that most clinical NLP systems are specific to an institution, which hinders their wider implementation. We showed that for the same task our models work across two very different sources. Moreover, the evaluation using MIMIC-III can lead to models being more commonly shared and compared.

### Results

For this preliminary investigation we report the results of our models on a small sample of radiology reports. For each condition we randomly sampled from the training set 50 reports predicted positive and 50 reports predicted negative. Without indicating the labels we asked a clinical oncologist to annotate the reports as a positive/negative instance. We report the results in Table 2. Overall, we achieved very high results – at least 84% precision and 88% recall. The models perform better for hydronephrosis, which can be due to the challenges we noted above for the brain metastasis model.

**Table 2:** Precision and recall results for the preliminary experiments

	Hydronephrosis		Brain metastasis	
	LTHT PPM	MIMIC-III	LTHT PPM	MIMIC-III
Precision	94%	93%	89%	84%
Recall	95%	96%	94%	88%

## Discussion

Results of this three-month pilot project for the task of clinical document retrieval were very promising. We took a simple, shallow NLP approach, which combined with the IBM Watson Content Analytics architecture and functionalities allowed us to process millions of documents in a relatively short period of time (e.g. a subset of 50,000 documents used to develop initial rule sets took approx. 30 min to process). We tested our models on a small manually annotated sample and obtained high precision and recall values. We made the following observations during the study.

### Generalisability

We found that the models worked best when they were task-specific. In the case of hydronephrosis the condition keywords are both cohesive and specific, which allows us to accurately identify the condition mentions in text. For the brain metastasis case study we observed that the condition keywords are often separated, sometime across sentences. They are also fairly generic and ambiguous keywords, which makes finding relevant documents difficult. In terms of extending our methods to other conditions, we hypothesise that as long as the new condition follows the same patterns as an implemented model, then it should be the simple case of replacing the condition dictionary and running the model for the same type of report (e.g. a condition with very specific keywords could reuse the hydronephrosis model). Even for the more challenging cases like brain metastasis, the model might be re-implemented for similar conditions (e.g. lung metastasis), where only the tumour location dictionary would need to be replaced. More broadly, the negative instance indicators can be reused across conditions. However, this probably would not hold for more distinct types of clinical reports, e.g. negation in radiology is very explicit (*no*, *no evidence of*, *without*), but in clinical notes implicit negation could be present as well (e.g. patient *denies* feeling numbness). Finally, the dictionaries we used were only developed and evaluated within a single institution. When applied to other collections of radiology reports, our dictionaries might account for the standard set of terms and their spelling, but there might be some further institution- or country-specific terms or acronyms which would need to be added.

### Handling difficult cases

When conducting the error analysis we noted some cases which present a significant challenge for the automatic processing of clinical reports (cf. Table 3). The first case can be ambiguous depending on the task setting – if looking at condition incidence in reports, then it should be treated as negative according to our definition, however if looking at patient incidence, then this would be considered as a positive cases. Clear task definitions based on requirements are needed to avoid false positives. The second case is ambiguous due to the radiologist’s phrasing – “without frank hydronephrosis”, in this case a system which values recall over precision (e.g. if the goal was to find any and all potentially positive cases for quality assurance) would need to treat this as a positive case. Although rare, these cases still need to be accounted for and clinical domain experts should decide whether such a case should be considered positive or negative. Finally, the third case shows how modality (“raised the possibility”) can lead to false positives. For such cases we introduced an optional rule, whereby the last condition mention in a report is treated as the one to be used for indexing. Overall, we recommend a thorough error analysis, which can point to such cases and thus help to refine the models.

**Table 3:** Examples of challenging cases of hydronephrosis incidence

“Reason: Evaluate for effusion; Admitting Diagnosis: HYDRONEPHROSIS”	
“The right kidney is considerably lower in location than the left with slight pelviectasis but without frank hydronephrosis”	
“FINDINGS: […] Initial images raised the possibility of mild bilateral hydronephrosis; however, repeat imaging with a different probe and in different positions suggests that this is likely fat hypertrophy within the renal sinus bilaterally. […] IMPRESSION: No definite hydronephrosis.”	

### Technical notes about the system

The processing speeds were monitored throughout the project, however due to technical problems with the server infrastructure, the performance of the system varied. We noted that on average simple indexing (i.e. tokenisation which enabled free-text search) of documents was about 200 documents per second. More sophisticated indexing which included multiple dictionaries and rule sets took on average about 30 documents per second. We also noted that the data ingestion is quite sensitive to the formatting, which necessitates robust data cleaning. This is a considerable issue, since in our experience clinical text data does not have high-quality formatting. Similarly, the data export in CSV format failed, because there was no way to protect the text data, which resulted in misaligned data. Exports in other formats had no such issues, so post-processing to derive data in CSV format is possible. Finally, we noted the advantage of the parallel architecture implemented in the system, which allows for simultaneous importing, parsing and searching of documents, resulting in significant time savings.

### Limitations

This feasibility study aimed to provide feedback to NHS stakeholders and IBM about advantages/disadvantages and issues in implementing a commercial text analytics system for use with clinical free-text reports. Our focus was on the technical infrastructure needed to set up a commercial system that could use NHS data in a way that preserves best data governance practices. We established that this is possible through the use of data safe facilities between the NHS trust and the IRC however most hospitals do not have access to such a research environment. A limitation of our study is therefore that we conducted our case study in a research environment connected to clinical systems rather than in the live clinical system itself. This was for the obvious reason that we needed to avoid any possibility of the research impacting on the care of patients within the hospital, for example by data processing demands slowing down the live servers. Similarly, we used the full set of available historical data collected over many years recognising that there may be temporal drift in the use of language and terms over the 10 year time period. Our case study was limited to a small number of report types and clinical conditions, whereas an actual implementation would be a part of a larger pipeline. In addition to technical factors of such integration, there are also the significant issues of acceptability and usability by NHS staff.

## Conclusions

In this paper we presented the results and observations from a feasibility study on implementing a commercial text analytics system – IBM Watson Content Analytics – with free-text clinical reports obtained from the NHS. We first presented an overview of the IBM Watson Content Analytics architecture. We then described our methods and results using this architecture for two examples from radiology: hydronephrosis and brain metastasis. The IBM Watson Content Analytics system allowed us to build a rule-based NLP model and apply it to millions of documents. Although the system scaled up to large datasets, it still required considerable manual input to create dictionaries and rule sets. However, we found that although we need to customise the models for each task, some resources and models could be reused between the two datasets with only some adjustments. We believe it is significant that our task-specific models generalise across radiology reports from two very different countries (UK and USA) for the two conditions we chose.

Our approach does allow us to reflect on the remaining implementation challenges. IBM Watson Content Analytics has the functionality to continuously ingest new data as a live data stream and output results through its user interface or as an output data stream. We therefore envisage a live data stream of radiology reports into the text analytics engine which returns a stream of coded results back into the EHR in the form of coded events and as alerts. In common with many other EHRs, LTHT’s PPM system has a) the ability to store and display such coded event data so that it can be reviewed by clinicians at point of care and b) an alerts system that can be set to notify clinicians of events of concern. Arguably, now that the text processing rules have been developed there is a case that they could be implemented directly as programme code within the EHR itself and this would reduce costs and improve processing speed. However, processing speeds of 30 documents per second suggests that system will already perform significantly faster than both human interpretation and the rate of production of new reports. In addition, IBM Watson Content Analytics provides a rich set of features for the continuous evaluation and improvement of NLP text analytics rules and we envisage a learning health system [26] where there is an ongoing requirement for text analytics rules that are extended, refined and improved on to account for temporal drift and make continuous improvements in patient safety. The implementation of user interface changes and alerts to achieve this within clinical settings is, in itself, a complex domain and is beyond the scope of this study.

In our study we found it was feasible to use IBM Watson Content Analytics within the UK NHS but there are other good large-scale text analytics systems available such as cTAKES, GATE and other commercial platforms. We note that IBM Watson Content Analytics uses the UIMA content analytics standards to support the transfer of dictionaries and models between different systems and we believe this presents an opportunity to separate the discussion on tools and rules from the discussion on implementation. We therefore envisage further work along three lines of investigation: a) what tools and techniques are best for generating and refining healthcare text analytics dictionaries and models? b) what are the optimum rules, dictionaries and models for each clinical condition given the inevitable trade-off between global generalisability and local language, culture and custom as clinical text evolves over time? and c) how best to implement text analytics rules, dictionaries and models within real time live clinical environments? Our feasibility study suggests that healthcare text analytics is sufficiently advanced to be operationalised within EHR solutions. The biggest challenge ahead is likely to be the design and implementation of real time processing solutions which will demonstrate the promised step change in clinical safety.

## Data Availability

Not applicable.

## Abbreviations

EHR: Electronic Health Record
IR: Information Retrieval
IRC: Integrated Research Campus
LTHT: Leeds Teaching Hospitals Trust
NHS: National Health Service
NLP: Natural Language Processing
PPM: Patient Pathway Manager
UIMA: Unstructured Information Management Architecture
UMLS: Unified Medical Language System

## Footnotes

1 https://www.ibm.com/support/knowledgecenter/en/SS5RWK [Accessed 7th June 2018]

2 https://www.ibm.com/smarterplanet/us/en/ibmwatson/developercloud/ [Accessed 7th June 2018]

3 See an example here: http://alt.qcri.org/semeval2015/task14/ [Accessed 7th June 2018]

4 https://mimic.physionet.org/ [Accessed 7th June 2018]
